# Catalpol induces autophagy and attenuates liver steatosis in ob/ob and high-fat diet-induced obese mice

**DOI:** 10.18632/aging.102396

**Published:** 2019-11-07

**Authors:** Huihui Ren, Dan Wang, Lu Zhang, Xiaonang Kang, Yaling Li, Xinrong Zhou, Gang Yuan

**Affiliations:** 1 Department of Endocrinology, Tongji Hospital, Tongji Medical College, Huazhong University of Science and Technology, Wuhan, Hubei 430030, P.R. China

**Keywords:** catalpol, nonalcoholic fatty liver disease, autophagy, AMPK, obesity

## Abstract

Impaired autophagy has been implicated in the pathogenesis of nonalcoholic fatty liver disease. Catalpol (CAT), a bioactive compound from *Rehmannia* (Di Huang) *glutinosa*, is known to ameliorate insulin resistance and the histological NAFLD spectrum in obese mice. Here, we investigated the effects of CAT on hepatic steatosis and autophagy in ob/ob and high-fat diet-induced obese mice, as well as in hepatocytes. In ob/ob mice, CAT reduced liver weight, liver triglyceride and cholesterol content, and hepatic lipogenic enzyme levels and increased fatty acid oxidase levels. In addition, CAT administration increased LC3-II levels and decreased SQSTM1/P62 levels in ob/ob mice. Similar effects on hepatic steatosis and autophagy were observed in high-fat diet-induced mice after administration of CAT. Additionally, we found that CAT stimulated AMPK and increased nuclear translocation of transcription factor EB (TFEB) in obese mice and hepatocytes. Inhibition of AMPK completely blocked the effects of CAT on TFEB nuclear localization, hepatic autophagy, and liver steatosis. These findings revealed that diminished AMPK/TFEB-dependent autophagy is involved in the pathogenesis of liver steatosis in obesity, and that CAT might be a novel therapeutic candidate for treatment of this condition.

## INTRODUCTION

The incidence of nonalcoholic fatty liver disease (NAFLD), which often accompanies obesity, is on the rise worldwide. NAFLD affects up to 20% of the general population, and up to 70% of patients with type 2 diabetes [1, 2]. Its clinicopathological features range widely from simple steatosis to nonalcoholic steatohepatitis (NASH), which may be accompanied different degrees of fibrosis and cirrhosis and may ultimately lead to hepatocellular carcinoma [3, 4]. NAFLD is strongly associated with obesity, insulin resistance, and subclinical systemic inflammatory state; lifestyle modification, weight loss, insulin-sensitizing agents, and bariatric surgery are therefore commonly recommended treatments [1]. However, there are no safe and effective pharmacologic therapies available for the treatment of NAFLD.

Catalpol (CAT), an iridoid glucoside isolated from the root of Rehmannia glutinosa that has anti-oxidant [5] and anti-inflammatory [6] effects, can counteract anti-insulin resistance in an animal model of diabetes [7]. CAT reduced obesity- and diet-induced insulin resistance and inflammation, which are both major contributors to NAFLD pathogenesis [6, 8]. Yan et al. [9] also reported that CAT ameliorated hepatic steatosis as well as insulin resistance in HFD/streptozocin (HFD/STZ)-induced diabetic mice. Moreover, in STZ-fat-diabetic rats, CAT treatment markedly reduced serum cholesterol (TC) and triglyceride (TG) levels [10]. However, the effects of CAT on NAFLD remain unknown.

Autophagy is an important cellular homeostatic processes in which cytoplasmic components, including damaged organelles and proteins, are transported to lysosomes for degradation [11]. Accumulating evidence indicates that autophagy is necessary for maintaining lipid metabolism homeostasis [12, 13]. Lipid droplets are recognized by autophagosomes and eventually degraded by lysosomes through a process termed lipophagy [14]. Dysregulation of autophagy might therefore contribute to liver fat accumulation, injury, inflammatory responses, fibrosis, and carcinogenesis, all of which are involved in the pathogenesis of NAFLD [15, 16]. Thus, we hypothesized that activation of autophagy might improve the histological NAFLD spectrum in HFD and ob/ob mice.

In addition, CAT has been reported to ameliorate inflammation and promote autophagy [17] and might therefore represent a novel treatment strategy for obesity-related metabolic disorders. Here, we investigated the effects of CAT on both hepatic steatosis in ob/ob and HFD-induced mice and on autophagy and lipid metabolism pathways.

## RESULTS

### CAT attenuated liver steatosis in ob/ob and HFD mice

**Figure 1:**
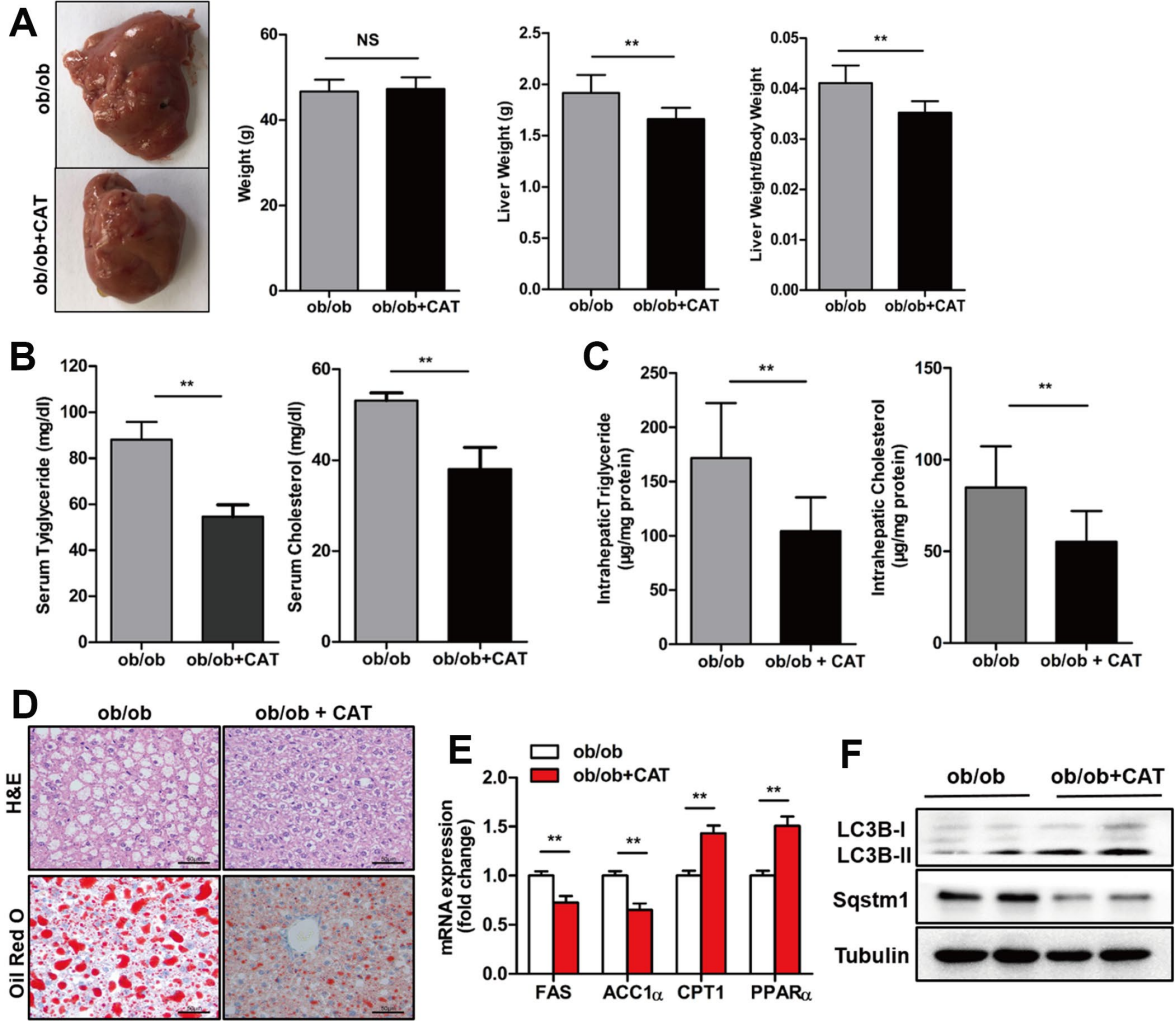
**CAT ameliorated hepatic steatosis in ob/ob mice.** Eight week-old ob/ob mice were treated with CAT (50 mg/kg/d) or vehicle by oral gavage for 4 weeks. (**A**) Gross images of liver tissue and changes in liver and body weights. (**B**) Serum TG and TC levels. (**C**) Liver TG and TC content normalized to total protein. (**D**) Representative photomicrographs of liver sections stained with H&E and Oil Red O. Scale bars: 50 μm. (**E**) mRNA expression levels of hepatic lipogenic genes ACC1α and FAS and fatty acid oxidation genes PPARα and CPT1. Data are expressed as fold-change relative to vehicle-treated ob/ob mice. (**F**) Representative western blot analysis of LC3-II, Sqstm1/P62, and Becn1 proteins. Unpaired two-sided t-tests were used for statistical comparisons to controls. ^*^P < 0.05, ^**^P < 0.01, ^***^P < 0.001 vs. vehicle-treated ob/ob mice.

To explore the effect of CAT on fatty livers, we administrated CAT or saline (control) to ob/ob and HFD mice intragastrically and analyzed liver/body weight ratios, liver tissues, and serum samples. In ob/ob mice, body weights were unchanged after CAT administration, but liver weight, liver/body weight ratios, serum TG and TC levels, and hepatic TG and TC content were all reduced (*P* <0.05) (Figure 1A–1C). H&E and Oil Red O staining indicated that hepatosteatosis also improved after CAT administration in ob/ob mice (Figure 1D). These data suggested that CAT alleviated liver steatosis, serum and liver lipid phenotype in obesity. Interestingly, CAT treatment downregulated expression of several hepatic lipogenic genes, such as ACC1α and FAS (*P* <0.05), and upregulated fatty acid oxidation genes, such as PPARα and CPT1 (*P* <0.05) (Figure 1E).

In addition, after CAT administration, protein levels of LC3-II, a well-established marker of autophagy induction, increased, while levels of SQSTM1/p62 protein, which accumulates when autophagy is suppressed, decreased (Figure 1F). These results indicate that CAT might prevent liver steatosis by inducing autophagy.

**Figure 2:**
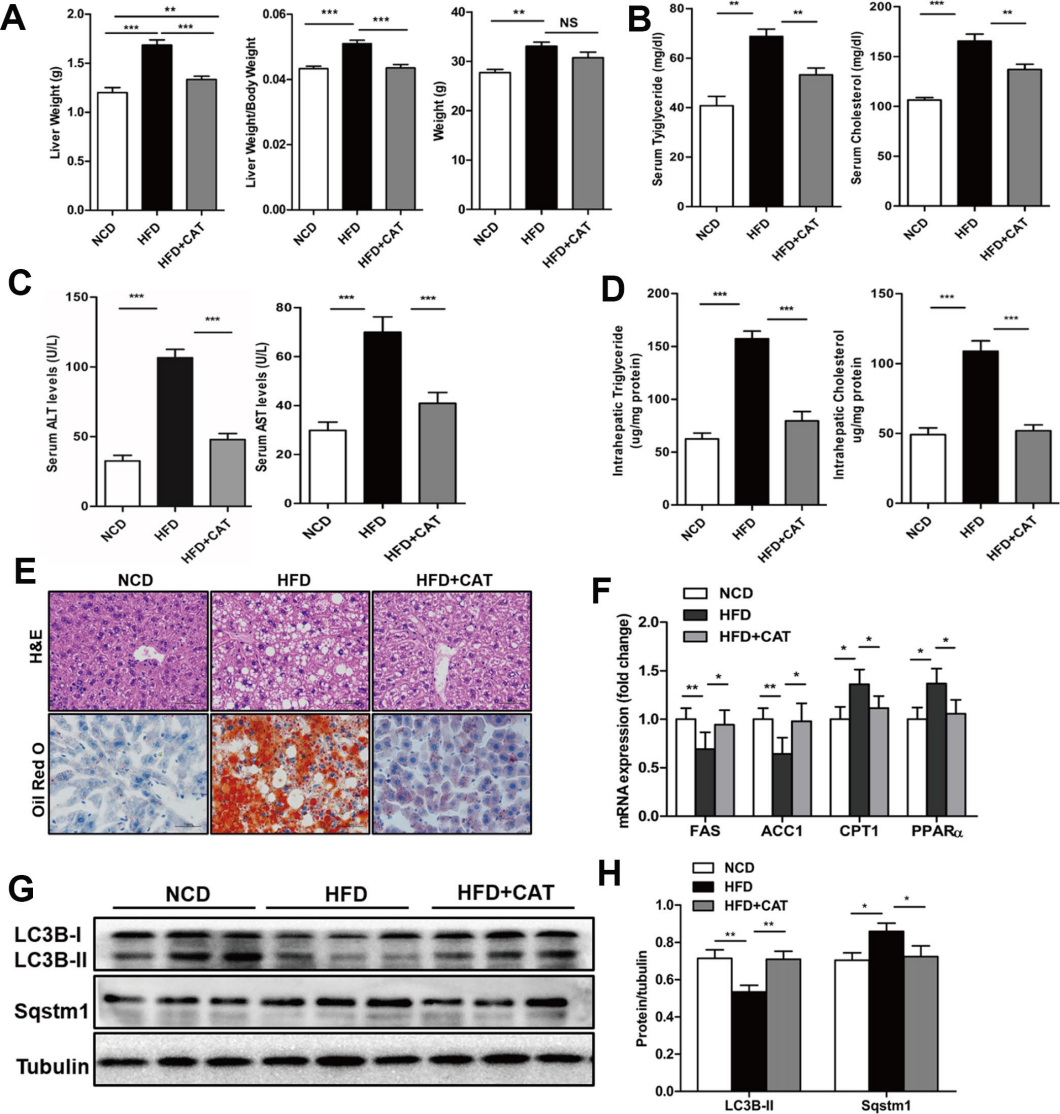
**CAT ameliorates liver steatosis in HFD-fed mice.** Mice were treated with CAT (50 mg/kg/d) or vehicle by oral gavage for 4 weeks. (**A**) Gross images of liver tissue and changes in liver and body weights. (**B**) Serum TG and TC levels. (**C**) Serum ALT and AST levels. (**D**) Liver TG and TC content normalized to total protein. (**E**) Representative photomicrographs of liver sections stained with H&E and Oil Red O. Scale bars: 50 μm. (**F**) mRNA expression levels of hepatic lipogenic genes ACC1α and FAS and fatty acid oxidation genes PPARα and CPT1. Data are expressed as fold-change relative to NCD mice. (**G**) Representative western blot analysis of LC3B-II and Sqstm1/P62. (**H**) LC3B-II and P62 band densities were normalized to tubulin. Means ± SD were calculated from three independent experiments. One-way ANOVAs with Tukey post-hoc tests were performed. n =5 per group. ^*^P < 0.05, ^**^P < 0.01, ^***^P < 0.001.

Next, we examined whether CAT also had beneficial effects on liver steatosis in a HFD-induced obesity mice model. Remarkably, liver weights, liver/body weight ratios, serum TG, TC, ALS, and ALT levels, and liver TG and TC content all decreased after CAT administration in HFD mice (*P* <0.05) (Figure 2A–2E). Furthermore, as was the case in ob/ob mice, ACC1α and FAS mRNA expression decreased, while PPARα and CPT1 expression increased, after CAT administration in HFD mice (Figure 2F). LC3II protein levels also increased, while SQSTM1/p62 protein levels decreased, after CAT administration in HFD mice (Figure 2G, 2H). Because free fatty acid (FFA)-induced lipotoxicity and resulting cell death are important features of the pathogenesis of NAFLD [18], we next investigated the effect of CAT on caspase-3 (CASP3) activity in HFD mice. Indeed, HFD was cytotoxic to hepatocytes, and CAT significantly attenuated this cytotoxicity (Supplementary Figure 1). Together, these results suggest that CAT ameliorates HFD-induced hepatic steatosis by activating autophagy.

### CAT induced autophagy in hepatocytes

**Figure 3:**
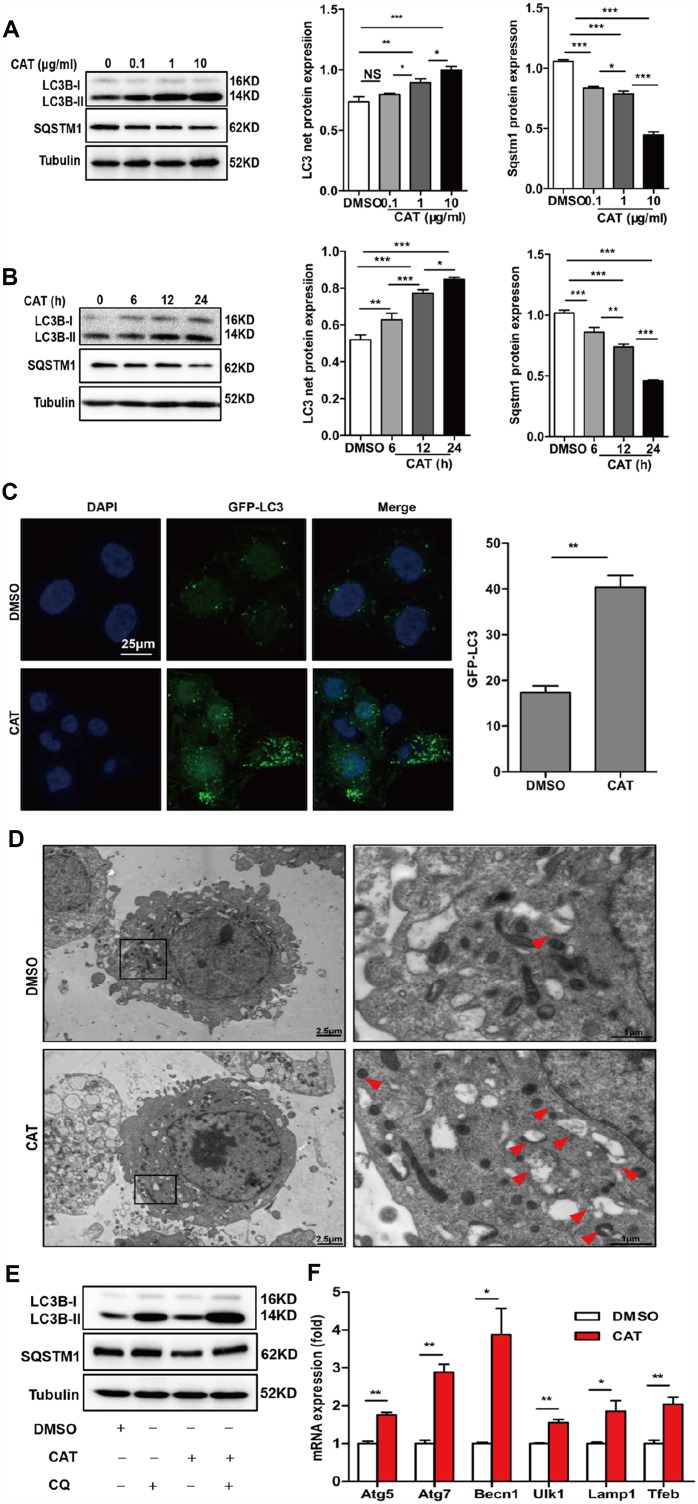
**CAT induced autophagy in hepatocytes.** (**A**) Dose-dependent induction of autophagy by CAT. Representative immunoblot analysis of LC3-II and SQSTM1 expression in lysates from HepG2 cells that were treated with CAT or control solvent (dimethyl sulfoxide, DMSO) at the indicated concentrations for 24 h. (**B**) Time-dependent induction of autophagy by CAT. Immunoblot detection of LC3-II and SQSTM1 expression in HepG2 cells treated with CAT (10 μg/mL) or DMSO for the indicated time. (**C**) HepG2 cells were transduced with an GFP-LC3 plasmid for 48 h and then treated with CAT (10 μg/mL) for 24h. Cells were observed by fluorescence microscopy to evaluate the number expressing GFP-LC3. (**D**) Representative electron microscopic pictures of HepG2 cells treated with CAT for 24 h. Arrows indicate autophagosomes. (**E**) Immunoblot analysis of LC3-II and SQSTM1 expression in HepG2 cells treated with 10 μg/mL CAT for 24 h in the absence or presence of 50 mM chloroquine (CQ) for the last 2 h. (**F**) qPCR analysis of autophagy-related and lysosomal genes in CAT-treated HepG2 cells. Means ± SD were calculated from three independent experiments. ^*^P < 0.05, ^**^P < 0.01.

Previous reports have suggested that autophagy plays a crucial role in hepatic steatosis and that it is suppressed in the livers of NASH patients and HFD mice. To evaluate the effects of CAT on autophagy, hepatic steatosis, and the underlying mechanisms, HepG2 cells were exposed to different concentrations (0.1, 1, or 10 μg/mL) of CAT for 24 h or treated with 10 μg/mL CAT for 6, 12, or 24 h. CAT treatment increased LC3-II levels and decreased SQSTM1/p62 levels in a dose- and time- dependent manner (Figure 3A, 3B). We next evaluated the effect of CAT on autophagy using fluorescence microscopy in GFP-LC3 transduced hepatocytes. LC3-II levels markedly increased in response to 24 h of CAT administration (Figure 3C). Additionally, electron microscopy revealed that more autophagic vacuoles formed in hepatocytes treated with CAT compared to controls (Figure 3D). To investigate the effects of CAT on autophagy flux, HepG2 cells were treated with CAT for 24 h with or without CQ (50 μM). Following exposure to CAT, LC3B-II levels increased, and SQSTM1/p62 levels decreased, in hepatocytes. CQ alone significantly increased LC3-II and SQSTM1/p62 levels, while coadministration with CAT further increased LC3-II levels and decreased SQSTM1/p62 accumulation (Figure 3E). Consistent with that result, GFP-LC3-II expression levels decreased, while free-GFP expression levels increased, in hepatocytes treated with CAT (Supplementary Figure 2). We also observed that CAT significantly upregulated autophagy-related genes (including Atg7, Atg5, Becn1, Ulk1, and Lamp1) and TFEB in HepG2 cells (Figure 3G). Together, these data indicate that CAT enhanced autophagy in hepatocytes.

### CAT induced autophagy via the AMPK-TFEB pathway

**Figure 4:**
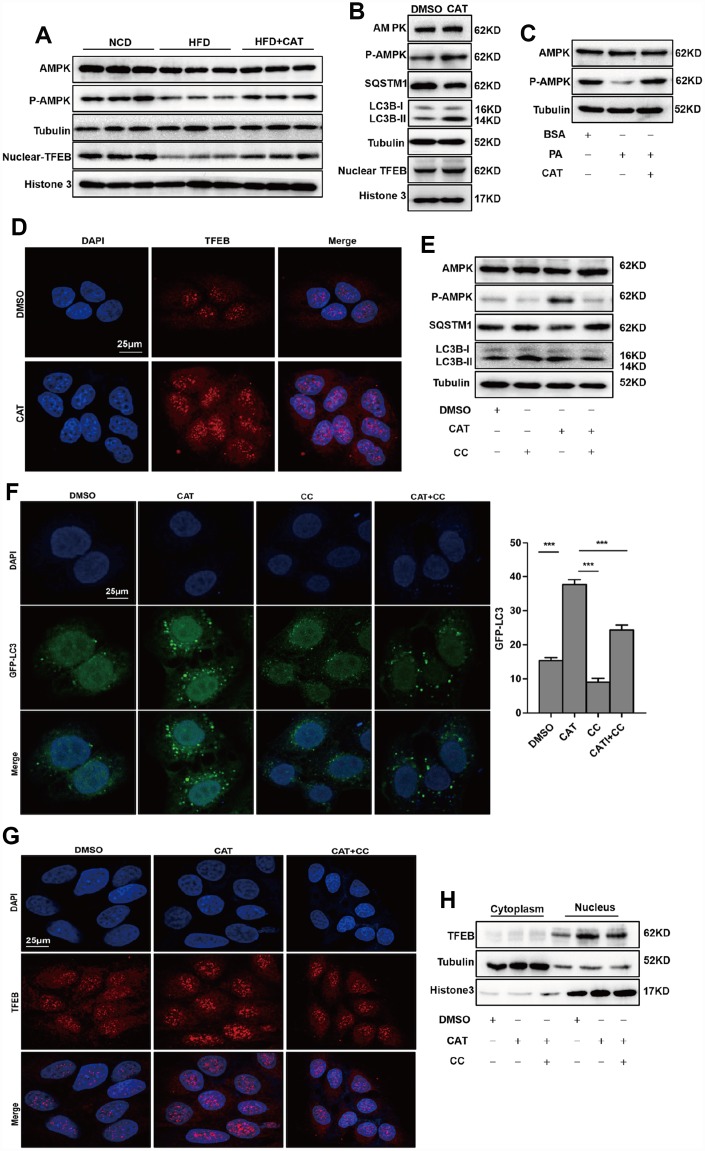
**CAT induced autophagy via the AMPK-TFEB pathway.** (**A**) Immunoblot detection of TFEB, AMPK, and AMPK phosphorylation levels in livers of mice. (**B**) Immunoblot detection of LC3-II, SQSTM1, TFEB, AMPK, and AMPK phosphorylation levels in HepG2 cells treated with CAT (10 μg/mL) for 24 h. (**C**) Immunoblot analysis of AMPK and p-AMPK levels in HepG2 cells treated with 0.3 mM palmitate (PA) and 10 μg/mL CAT for 24 h. (**D**) Fluorescence microscopy images of nuclear TFEB in HepG2 cells treated with 10 μg/mL CAT for 24 h. Scale bars: 25 μm. (**E**) Immunoblots of LC3-II, SQSTM1, AMPK, and AMPK phosphorylation in HepG2 cells treated with CAT (10 μg/mL) and Compound C (CC, 10 μM) for 24 h. (**F**) Numbers of GFP-LC3 in HepG2 cells expressing GFP-LC3 after treatment with or without CAT (10 μg/mL) and CC (10 μM) for 24 h were evaluated using fluorescence microscopy. Scale bars: 25 μm. (**G**, **H**) Fluorescence microscopy images and Immunoblot analysis of nuclear TFEB in HepG2 cells treated with 10 μg/mL CAT and Compound C (CC, 10 μM) for 24 h. Scale bars: 25 μm. ^***^P < 0.001.

Previous studies report that AMPK activates autophagy and reduces lipid accumulation *in* *vivo* and *in* *vitro* [19, 20]; we therefore evaluated the effects of CAT on AMPK activation in HFD-fed mice (Figure 4A). Notably, CAT induced AMPK phosphorylation and activated autophagy in HepG2 cells (Figure 4B). In addition, AMPK phosphorylation, but not levels of AMPK itself, decreased significantly in the presence of PA and increased after CAT treatment (Figure 4C). TFEB activates the transcription of genes that promote lysosomal biogenesis and regulates autophagy upon translocation to the nucleus. We therefore examined whether CAT treatment activated autophagy by promoting translocation of TFEB. Western blot analysis showed that CAT increased nuclear TFEB translocalization in hepatocytes (Figure 4A, 4B. Nuclear TFEB levels in hepatocytes after CAT administration as determined by fluorescence analyses were consistent with these results (Figure 4D). We then tested whether AMPK was required for CAT-induced autophagy and nuclear TFEB translocation. Inhibition of AMPK by CC reduced autophagic activity as evidenced by decreased LC3-II levels and increased SQSTM1/p62 levels (Figure 4E) and by a significant decrease in the number of GFP-LC3-positive vesicles (Figure 4F). Furthermore, fluorescence analyses and Western blots indicated that CC abrogated CAT-induced nuclear translocation of TFEB (Figure 4G, 4H). Collectively, these findings indicate that CAT induces autophagy via an AMPK/TFEB-related pathway.

### CAT ameliorated liver steatosis via AMPK-induced autophagy

**Figure 5:**
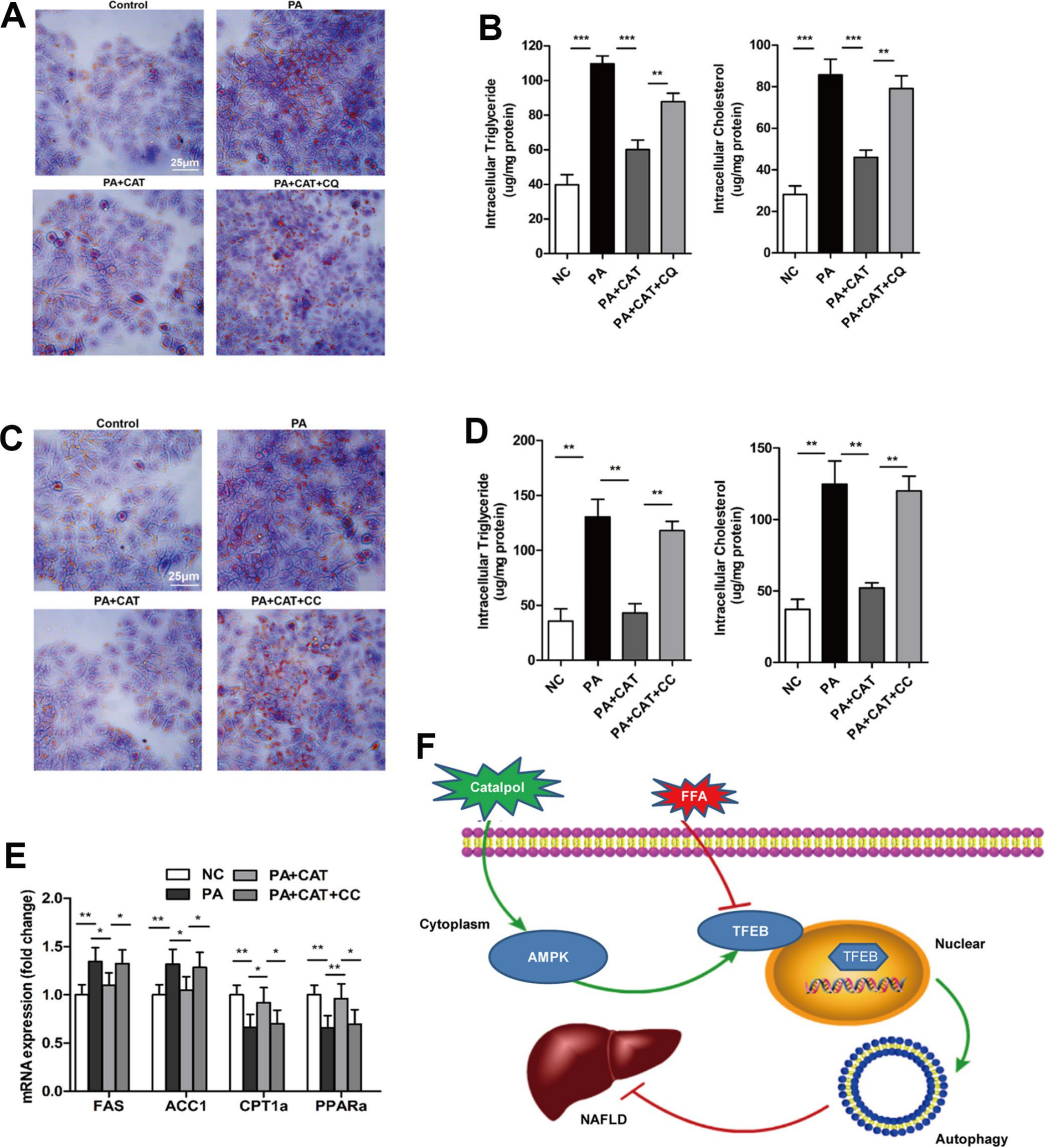
**CAT ameliorated liver steatosis by inducing autophagy via AMPK.** Cells were treated with 0.3 mM PA and 10 μg/mL CAT for 24 h in the presence or absence of 50 mM chloroquine (CQ) for 2 h. HepG2 cells were stained with Oil Red O (**A**), and intracellular TG and TC were quantitatively analyzed (**B**). Scale bars: 25 μm. HepG2 cells treated with 0.3 mM PA, 10 μg/mL CAT for 24 h, and 10 μM compound C (CC) for 24 h. Cells were stained with Oil Red O (**C**), and intracellular TG and TC were quantitatively analyzed (**D**). Scale bars: 25 μm. (**E**) Expression of hepatic lipogenic genes ACC1α and FAS and fatty acid oxidation genes PPARα and CPT1 was quantitatively analyzed (n=3). (**F**) A schematic illustration demonstrating the role of CAT in NAFLD and the effect of CAT on autophagy. ^*^P < 0.05, ^**^P < 0.01, ^***^P < 0.001.

To determine whether the protective effects of CAT against liver steatosis were autophagy-dependent, an *in* *vitro* model of NAFLD was established by treating HepG2 cells with PA for 24 h (Supplementary Figures 3A, 4B). Flow cytometry indicated that apoptosis levels increased significantly in cells treated with PA (Supplementary Figure 3C). Lipid staining and liver lipid content analyses showed that CAT significantly attenuated PA-induced lipid accumulation in hepatocytes; this protective effect of CAT decreased in the presence of CQ (Figure 5A, 5B). We then examined whether the inhibitory effects of CAT on lipotoxicity-mediated apoptosis was dependent on autophagy in hepatocytes. CAT treatment attenuated lipoapoptosis, as evidenced by decreased cleaved caspase-3 and PARP expression levels, but these effects were eliminated by co-treatment with CQ (Supplementary Figure 4A). These results were confirmed by flow cytometry analysis (Supplementary Figure 4B). Together, these data indicate that CAT inhibits lipid accumulation and lipoapoptosis by promoting autophagic flux.

Next, we investigated whether the AMPK-dependent pathway identified above was involved in CAT-dependent reversal of liver steatosis. CAT markedly reduced PA-induced intracellular lipid accumulation, and co-treatment with CC attenuated this effect, indicating that the protective effects of CAT against hepatic steatosis are mediated by the AMPK pathway (Figure 5C, 5D). More importantly, incubation of hepatocytes with CC completely abrogated CAT-induced upregulation of the fatty acid metabolism genes FAS, ACC1, CPT1, and PPARα (*P* <0.05) (Figure 5E).

## DISCUSSION

In the present study, we explored new potential therapeutic effects of CAT, which can enhance autophagic activity and ameliorate hepatic steatosis in obese mice. The anti-steatosis effects of CAT may result from activation of AMPK via phosphorylation in liver cells, which in turn increases nuclear translocation of TFEB (Figure 5F). Furthermore, inhibition of AMPK phosphorylation blocked the effects of CAT. Our data indicate that CAT, a novel autophagy enhancer, might be an effective treatment for obesity-associated hepatosteatosis in experimental animal models.

Autophagy is a highly adaptive catabolic process which can provide energy for cells during nutrient deprivation or starvation to maintain cellular metabolism homeostasis, and impairments of autophagy may be implicated in the development of metabolic disorders [21]. NAFLD is characterized by widespread dysregulation of metabolic processes, including the suppression of autophagy in the liver [22]. It is likely that increased autophagy may protect against steatosis. For example, Lin et al. [23] found that pharmacological promotion of autophagy by rapamycin and carbamazepine protected against fatty liver pathologies by regulating lipid metabolism, reducing triglyceride levels, and improving insulin sensitivity in mice with HFD-induced obesity. However, pharmaceutical agents that promote autophagy may not always be practical treatments in humans due to side effects [24]. For example, rapamycin causes adverse effects in the immune system in humans. 3-methyladenine (3-MA), which induces autophagy by inhibiting Class III PI3K activity, also has additional undesirable off-target effects. Developing pharmacological agents that specifically activate autophagy to control hepatic steatosis remains challenging [25]. For that reason, we have explored other novel therapeutic approaches that might promote autophagy in NAFLD, including traditional Chinese medicine.

CAT, an iridoid glycoside isolated from Rehmannia, has beneficial effects in metabolic disorders [5, 6, 9]. CAT resulted in reduced body weight, decreases in serum triglycerides and liver enzyme levels, and improvements in insulin resistance in diabetic mice, linking CAT to the improvement of NAFLD [26]. Additionally, CAT can alleviate hepatic steatosis in obese mice by counteracting anti-oxidative stress and anti-insulin resistance [9]. Despite the multiple metabolic effects of CAT, its role in the development of NAFLD is not yet completely understood. The current study confirmed that CAT significantly improved hepatic steatosis in mouse models of obesity. Moreover, we investigated effects of CAT on autophagic activity and lipid metabolism in a mouse model of NAFLD and a cellular model of hepatocytic steatosis. The lysosomal inhibitor experiments performed here further confirmed the autophagy-dependent effects of CAT. A previous report that CAT protected against myocardial ischemia by promoting mitophagy also demonstrated a connection between CAT and autophagy [5]. Thus, CAT may be a negative regulator in obesity-related hepatic steatosis in vivo.

In this study, we also identified the mechanism by which CAT regulates autophagy. AMPK plays essential roles in cellular and organismal metabolism, as well as in a variety of metabolic diseases [27]. Activation of AMPK results in several beneficial metabolic effects in Type 2 diabetes and metabolic syndromes [28]. AMPK also plays critical roles in coordinating growth, autophagy, and metabolism [29]. In the present study, we observed that CAT significantly increased AMPK phosphorylation in HepG2 cells. Furthermore, compound C, an AMPK inhibitor, abolished CAT-induced improvement of liver steatosis and autophagy activation. These data suggested that AMPK was indispensable for the beneficial effects of CAT on hepatic steatosis, and AMPK-dependent autophagy might be an important mechanism by which CAT modulates energy metabolism.

Recent studies have revealed a novel role for transcription factors in the regulation of autophagy. The bHLH-leucine zipper transcription factor TFEB acts as a master transcription regulator of genes related to autophagy and lysosome biogenesis. It is involved in several key steps of the autophagic process, including autophagosome formation, autophagosome-lysosome fusion, and lysosome-mediated degradation of the cellular constituents [30]. Previous studies reported that nuclear localization of TFEB significantly upregulated transcription of genes encoding autophagic and lysosomal proteins, ultimately resulting in increases in autophagosome and lysosome formation and autophagy in general [31]. In this study, we found that CAT significantly increased nuclear translocation of TFEB and upregulated autophagy-related genes in hepatocytes. In addition, inhibition of AMPK phosphorylation by CC prevented the CAT-induced TFEB nuclear translocation, suggesting that it was AMPK-dependent. Moreover, TFEB increases expression of the PGC1α and PPARα genes, thereby regulating lipid metabolism in the liver [32]. These findings indicate that CAT may induce autophagy and alleviate hepatosteatosis via its effects on the AMPK/TFEB pathway.

In conclusion, the results of this study suggest that CAT attenuates hepatic steatosis and lipotoxicity by inducing autophagy via AMPK activation and subsequent nuclear translocation of TFEB in a mouse model. CAT might therefore be a promising potential therapy for liver steatosis associated with obesity in animals, though its potential in human disease remains unproven.

## MATERIALS AND METHODS

### Reagents

The following chemicals were used: Dulbecco's Modified Eagle Medium (DMEM, Gibco), fetal bovine serum (FBS, Gibco), pre-stained protein markers from Fermentas (Thermo Fisher Scientific, 26616), palmitate (PA, Sigma-Aldrich, P9767), chloroquine (CQ; Sigma-Aldrich, C6628), Oil Red O (Sigma-Aldrich, O1391), CAT (98% purity, MedChem Express, HY-N0820), Compound C (CC, MedChem Express, HY-13418A).

### Mouse models and CAT treatment

Eight-week old male ob/ob mice were purchased from Beijing Huafukang Bioscience Co. Inc. (Beijing, China). Animals were maintained under a 12 h light-dark cycle with free access to food and water. The mice were acclimatized to laboratory conditions for 1 week before the study and then divided into two groups: the control group (n=5) received a daily oral gavage of the vehicle (100 μL of sterile saline), while the CAT group (n=5) received CAT at 100 mg/kg of body weight. Mice were sacrificed after 4 weeks of treatment, blood was collected via the tail vein, and liver tissues were immediately removed, weighted, and stored at -80°C for further analysis.

Seven-week old male C57BL/6 mice obtained from Beijing Huafukang Bioscience Co. Inc. (Beijing, China) were housed in plastic cages under same conditions described above. After a 1 week acclimatization period, they were divided into three groups: one group received normal chow diet (NCD), and the two other received a HFD (60 Kcal% fat, D12492, Research diet, New Brunswick, NJ, USA) for 12 weeks with or without 50 mg/kg/day CAT dissolved in saline via gastric gavage for the last 4 weeks (n=5 in each group). After starvation for 6 h, mice were anesthetized with pentobarbital (40 mg/kg body weight) and blood and livers were collected, processed, and stored at -80°C. All animal experiments were approved by the Institutional Animal Research Committee of Tongji Medical College, Huazhong University of Science and Technology.

### Cell culture and treatment

HepG2 cells were cultured with DMEM supplemented with 10% FBS at 37°C in 5% CO_2_. To establish a cellular model of NAFLD, HepG2 cells were treated with PA at a concentration of 0.3 mM for 24 h [33]. For the CAT treatment experiment, hepatocytes were exposed to CAT at a concentration of 10 μg/mL for 24 h. CQ (50 μM) was added for the last 2 h [23]. To study the effect of AMPK inhibition on translocation and phosphorylation of TFEB or autophagy, cells were treated with 10 μM CC for 24 h [20].

### Quantitative PCR analysis

Total RNA was isolated from HepG2 cells and liver tissues using Trizol reagent (Invitrogen, 15596018) according to the manufacturer’s protocol. Two micrograms of total RNA were reverse transcribed using a FastQuant RT kit (Tiangenbiotech, Beijing, China, KR106). qPCR analysis was performed using Fast SYBR^®^ Green Master Mix (Thermo Fisher Scientific (Waltham, MA, USA) on a StepOne Plus™ Real-Time PCR System (Applied Biosystems). The sequences of primers used are listed in Supplementary Table 1. Relative gene expression levels were normalized to GAPDH expression level and calculated using the 2^−ΔΔC^ method.

### Western blot analysis

Proteins were extracted from HepG2 cells and liver tissues. About 20-50 μg of protein were separated on 12% sodium dodecyl sulfate–polyacrylamide gels (SDS-PAGE), transferred to PVDF membranes, and then blocked with 5% skim milk solution in Tris buffered saline containing 0.1% Tween-20 for 1.5 h at room temperature. Western blotting was performed using the following primary antibodies: anti-sequestosome 1 (SQSTM1/P62, Santa Cruz Biotechnology, sc-28359), anti-LC3 (Sigma-Aldrich, L7543), anti-Tubulin (Santa Cruz Biotechnology, sc-73242), anti-TFEB (Abcam, ab220695), anti-AMPK (Santa Cruz Biotechnology, sc-74461), anti-Phospho-AMPKα (Thr172, Cell Signaling Technology, #50081), anti-Caspase-3 (Cell Signaling Technology, #9665), anti-PARP (Santa Cruz Biotechnology, sc-365315). Blots were detected using a chemiluminescence system (Bio-Ras Laboratories, Berkeley, CA, USA). Band intensities were densitometrically analyzed using ImageJ 1.8.0 Software.

### Transfection and immunofluorescence

HepG2 cells were transfected with GFP-LC3 plasmid kindly provided by Fuqing Hu (Huazhong University of Science and Technology, Wuhan, China) for 48 h using Lipofectamine 2000 (Invitrogen/Life Technologies). The cells were then incubated with either CAT (10 μg/mL) or CC (10 μM) for 24 h. To identify nuclear translocation of TFEB, HepG2 cells were exposed to CAT (10 μg/mL) for 24 h. For immunofluorescence, cells were fixed in 4% paraformaldehyde, permeabilized using 0.1% Triton X-100, and blocked by incubating in PBST with 10% goat serum and 3% BSA for 1 h at room temperature. Samples were then incubated with primary antibody overnight at 4°C followed by incubation with secondary antibodies for 3 h. Images were captured with a confocal microscope (LSM700; Carl Zeiss Inc., Oberkochen, Germany).

### Transmission electron microscopy

Liver samples or cells were fixed with 2% glutaraldehyde/paraformaldehyde overnight at 4°C, treated with 1% osmiumtetroxideina for 1 h at room temperature, and dehydrated in graded ethanol solutions according standard procedures. Samples were then embedded in Epon 812 (Electron Microscopy Sciences,100503–876) and sectioned using a Reichert Ultracut E microtome (Leica Microsystems, Buffalo Grove, IL, USA). EM images were acquired using a H7600 transmission electron microscope (Hitachi, Tokyo, Japan).

### Hepatic and cellular TG and TC measurement

Intracellular and intrahepatic TG and TC content was measured using commercial kits (Jiancheng Technologies Inc, Nanjing, China) according to the manufacturer’s recommended protocols.

### Histopathological examination

Liver sections were fixed in 4% paraformaldehyde, embedded in paraffin, and then stained using hematoxylin and eosin (H&E) for histological examination. To determine lipid droplet accumulation, frozen liver sections (8 μm) were incubated with freshly diluted Oil Red O staining solution for 10 min, washed, and then counterstained with hematoxylin for 5 min. Cells were fixed in 4% paraformaldehyde for 15 min, stained with Oil Red O staining solution for 10 min, washed with 60% isopropanol, and stained with hematoxylin for 5 min. The histological features of the samples were observed and imaged at 400 × magnification using light microscopy (Olympus, Japan).

### Biochemistry assay

Serum TC, TG, aspartate transaminase (AST), and alanine transaminase (ALT) were measured using a colorimetric diagnostic kit (Jiancheng Bioengineering Institute, Nanjing, China) according to the manufacturer’s instructions.

### Caspase 3 activity assay

Cell viability was assessed using a caspase 3 activity assay kit (Beyotime Institute of Biotechnology, Haimen, China) according to the manufacturer’s protocol. Liver tissues (5 mg) were homogenized in 100 μL lysis buffer. Next, 50 μL lysates were exposed to 10 μL of the caspase 3 substrate in assay buffer (total volume: 100 μL) for 120 min at 37°C. Optical density was measured using an ELISA reader at an absorbance of 405 nm.

### Flow cytometry

To measure lipoapoptosis, cells were cultured with either PA (0.3 μM) or CAT (10 μg/mL) for 24 h. CQ (50 μM) was added for the last 2 h, followed by staining with Annexin V and 7-amino-actinomycin D (7-AAD; BD Biosciences, Mississauga, ON); flow cytometry was then performed using a FACSCanto II (BD Biosciences, Mississauga, ON). Early-stage apoptotic cells are PE Annexin V-positive and 7-AAD-negative, while late-stage apoptotic cells are both PE Annexin V- and 7-AAD-positive.

### Statistical analysis

The data are expressed as mean ± SD. Unpaired two-sided t-tests were used for comparisons between two groups. One-way analysis of variance (ANOVA) with post hoc Bonferroni tests was used for comparisons between multiple groups. *P* values < 0.05 were considered statistically significant.

## Supplementary Material

Supplementary Figures

Supplementary Table 1
