# Supplementary material for: Catalpol induces autophagy and attenuates liver steatosis in ob/ob and high-fat diet-induced obese mice
Source: Aging (Albany NY). 2019 Nov 7;11(21):9461–77. doi: 10.18632/aging.102396 (PMC6874442; doi:10.18632/aging.102396)
Supplement: Supplementary Figures [file aging-11-102396-s002.pdf]

SUPPLEMENTARY FIGURES

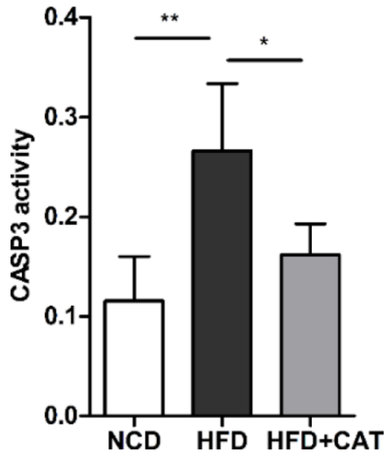

Supplementary Figure 1. CAT ameliorated lipoapoptosis in HFD mice. Mice were treated with CAT (50 mg/kg/d) or vehicle by oral gavage for 4 weeks. Caspase-3 (CASP3) activity was measured. *$P < 0.05$, **$P < 0.05$.

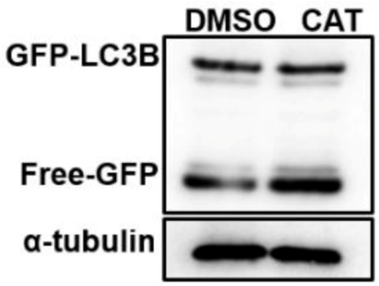

Supplementary Figure 2. CAT induced autophagic flux in hepatocytes. Immunoblot detection of the expression of GFP-LC3, and free-GFP in HepG2 cells treated with CAT (10 μg/ml) for 24H. GFP-LC3B expression levels decreased, and free-GFP expression levels increased in hepatocytes responded to CAT.

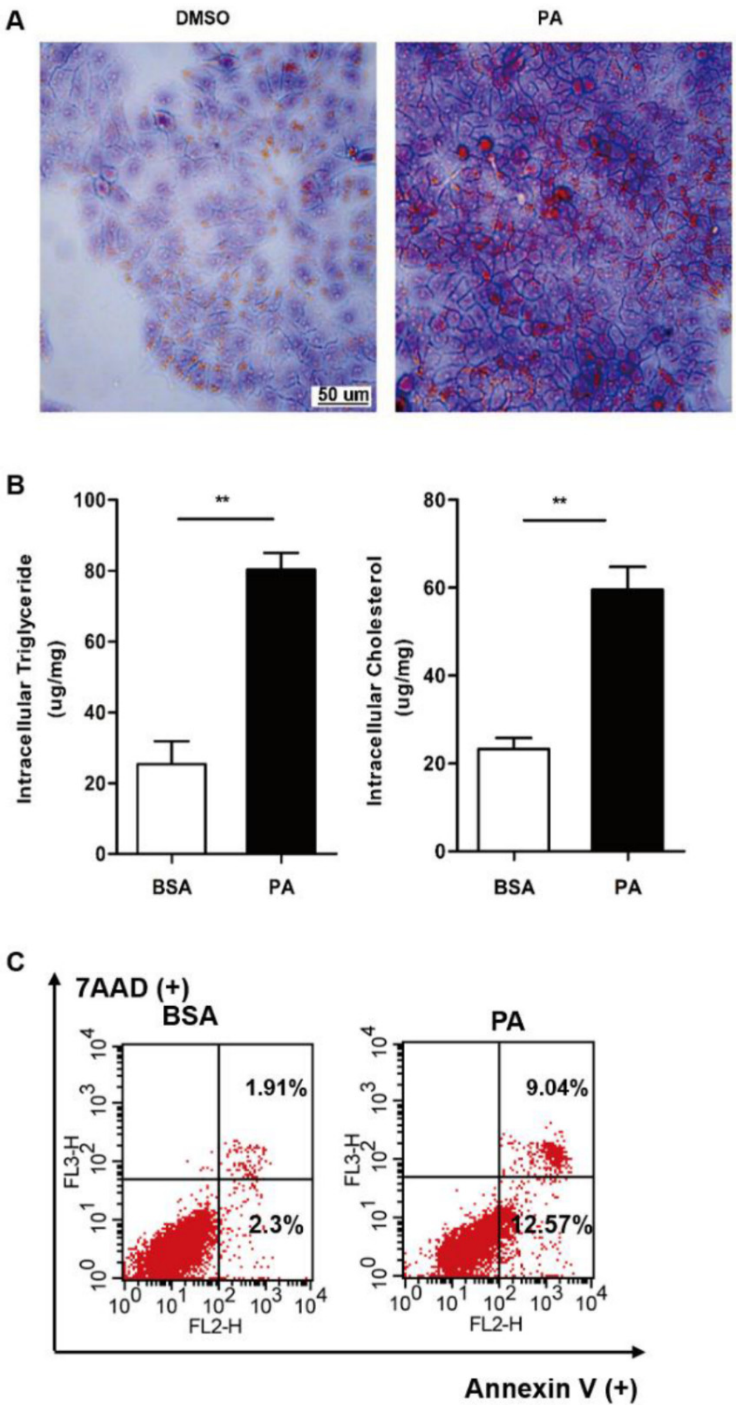

Supplementary Figure 3. PA induced liver steatosis and apoptosis in hepatocytes. Cells were treated with 0.3 mM PA for 24 h. HepG2 cells were stained with Oil Red O staining (**A**), and intracellular TG and TC was quantitatively analyzed (**B**). Scale bars: 25 µm. The apoptosis was determined by and flow cytometry (**C**).

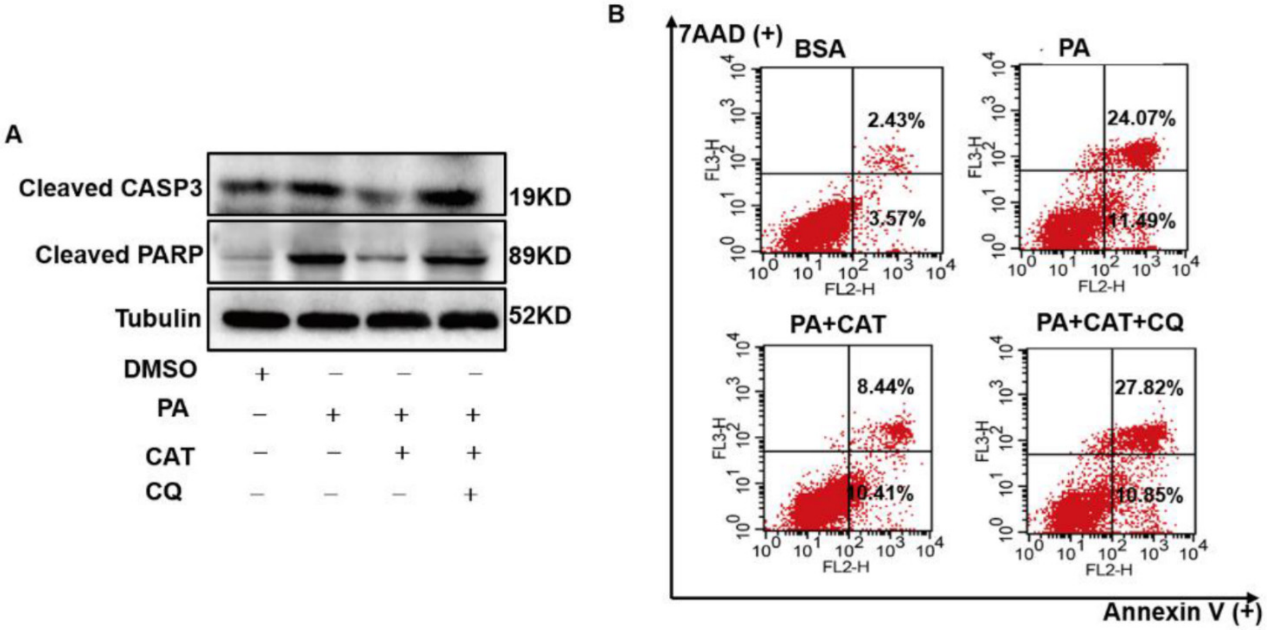

Supplementary Figure 4. CAT ameliorated lipoapoptosis in hepatocytes depending on autophagy. Cells were treated with 0.3 mM PA and 10ug/ml CAT for 24 h in the presence or absence of 50 mM chloroquine (CQ) for 2 h. The apoptosis was determined by immunoblot analysis (**A**) and flow cytometry (**B**)..
