# Supplementary material for: Catalpol induces autophagy and attenuates liver steatosis in ob/ob and high-fat diet-induced obese mice
Source: Aging (Albany NY). 2019 Nov 7;11(21):9461–77. doi: 10.18632/aging.102396 (PMC6874442; doi:10.18632/aging.102396)
Supplement: Supplementary Table 1 [file aging-11-102396-s001.pdf]

SUPPLEMENTARY TABLE

Supplementary Table 1. The primers used in the study.

Primer names	Sequences (5′->3′)
Human	
TFEB F	ACCTGTCCGAGACCTATGGG
TFEB R	CGTCCAGACGCATAATGTTGTC
ATG7 F	ATGATCCCTGTAACTTAGCCCA
ATG7 R	CACGGAAGCAAACAACTTCAAC
ATG5 F	AAAGATGTGCTTCGAGATGTGT
ATG5 R	CACTTTGTCAGTTACCAACGTCA
BECN1 F	GGTGTCTCTCGCAGATTCATC
BECN1 R	TCAGTCTTCGGCTGAGGTTCT
ULK1 F	AGCACGATTTGGAGGTCGC
ULK1 R	GCCACGATGTTTTCATGTTTCA
LAMP1 F	TCTCAGTGAACTACGACACCA
LAMP1 R	AGTGTATGTCCTCTTCCAAAAGC
PPARα F	ATGGTGGACACGGAAAGCC
PPARα R	CGATGGATTGCGAAATCTCTTGG
CPT1α F	ATCAATCGGACTCTGGAAACGG
CPT1α R	TCAGGGAGTAGCGCATGGT
FAS F	AGATTGTGTGATGAAGGACATGG
FAS R	TGTTGCTGGTGAGTGTGCATT
ACC1 F	ATGTCTGGCTTGCACCTAGTA
ACC1 R	CCCCAAAGCGAGTAACAAATTCT
GAPDH F	ACAACTTTGGTATCGTGGAAGG
GAPDH R	GCCATCACGCCACAGTTTC
Mouse	
Ppar F	TACTGCCGTTTTCACAAGTGC
Pparα R	AGGTCGTGTTCACAGGTAAGA
Cpt1α F	AGATCAATCGGACCCTAGACAC
Cpt1α R	CAGCGAGTAGCGCATAGTCA
Becn1 F	ATGGAGGGGTCTAAGGCGTC
Becn1 R	TGGGCTGTGGTAAGTAATGGA
Acc1 F	AATGAACGTGCAATCCGATTTG
Acc1 R	ACTCCACATTTGCGTAATTGTTG
Fas F	GCGGGTTCGTGAAACTGATAA
Fas R	GCAAAATGGGCCTCCTTGATA
Gapdh F	AGGTCGGTGTGAACGGATTTG
Gapdh R	TGTAGACCATGTAGTTGAGGTCA
